# VBP15, a novel anti-inflammatory and membrane-stabilizer, improves muscular dystrophy without side effects

**DOI:** 10.1002/emmm.201302621

**Published:** 2013-09-09

**Authors:** Christopher R Heier, Jesse M Damsker, Qing Yu, Blythe C Dillingham, Tony Huynh, Jack H Van der Meulen, Arpana Sali, Brittany K Miller, Aditi Phadke, Luana Scheffer, James Quinn, Kathleen Tatem, Sarah Jordan, Sherry Dadgar, Olga C Rodriguez, Chris Albanese, Michael Calhoun, Heather Gordish-Dressman, Jyoti K Jaiswal, Edward M Connor, John M McCall, Eric P Hoffman, Erica K M Reeves, Kanneboyina Nagaraju

**Affiliations:** 1 Center for Genetic Medicine Research, Children's National Medical CenterWashington, DC, USA; 2 ReveraGen BioPharmaRockville, MD, USA; 3 Endocrine Research Unit, The Canberra HospitalGarran, ACT, Australia; 4 Lombardi Comprehensive Cancer Center and Department of Oncology, Georgetown University Medical CenterWashington, DC, USA; 5 Department of Pathology, Georgetown University Medical CenterWashington, DC, USA; 6 Sinq SystemsSilver Spring, MD, USA; 7 Department of Integrative Systems Biology, George Washington University School of Medicine and Health SciencesWashington, DC, USA; 8 Center for Translational Science, Children's National Medical CenterWashington, DC, USA; 9 PharMac LLCBoca Grande, FL, USA

**Keywords:** anti-inflammatory, dystrophy, *mdx*, membrane injury, muscle

## Abstract

Absence of dystrophin makes skeletal muscle more susceptible to injury, resulting in breaches of the plasma membrane and chronic inflammation in Duchenne muscular dystrophy (DMD). Current management by glucocorticoids has unclear molecular benefits and harsh side effects. It is uncertain whether therapies that avoid hormonal stunting of growth and development, and/or immunosuppression, would be more or less beneficial. Here, we discover an oral drug with mechanisms that provide efficacy through anti-inflammatory signaling and membrane-stabilizing pathways, independent of hormonal or immunosuppressive effects. We find VBP15 protects and promotes efficient repair of skeletal muscle cells upon laser injury, in opposition to prednisolone. Potent inhibition of NF-κB is mediated through protein interactions of the glucocorticoid receptor, however VBP15 shows significantly reduced hormonal receptor transcriptional activity. The translation of these drug mechanisms into DMD model mice improves muscle strength, live-imaging and pathology through both preventive and post-onset intervention regimens. These data demonstrate successful improvement of dystrophy independent of hormonal, growth, or immunosuppressive effects, indicating VBP15 merits clinical investigation for DMD and would benefit other chronic inflammatory diseases.

## INTRODUCTION

Contraction-induced myofibre injury and inflammation are characteristic features of Duchenne muscular dystrophy (DMD), a fatal genetic muscle disease. We and others have demonstrated that the pro-inflammatory transcription factor NF-κB is active in dystrophin deficient muscle before symptom onset (Chen et al, 2005; Porter et al, 2002, 2003). Pharmacological glucocorticoids (prednisone, deflazacort) are standard of care in DMD, and we hypothesize their primary mechanism of action to be through anti-inflammatory activities via NF-κB pathways (Wissink et al, 1997). However, their harsh side effects in children greatly reduce patient adherence to glucocorticoid regimens and limit their therapeutic window. More general immunosuppressive compounds reduce inflammation in DMD but fail to increase patient strength in the same manner as glucocorticoids (Griggs et al, 1993; Kissel et al, 1993), while specific targeting of NF-κB increases strength in animal models (Grounds & Torrisi, 2004; Peterson et al, 2011). These data suggest that the specific mechanism by which glucocorticoids inhibit NF-κB is of particular importance to DMD treatment efficacy. Therapeutics that target this pathway in the absence of side effects may provide a substantial improvement in the treatment of DMD.

At the cellular level, dystrophin deficient muscles show increased susceptibility to stretch-mediated membrane instability and calcium dependent hyper-contracture (Bertorini et al, 1982; Yasuda et al, 2005), as well as increased oxidative stress (Disatnik et al, 1998; Rando et al, 1998). Glucocorticoids and other steroidal compounds are multi-mechanistic; in addition to binding hormonal receptors they can interact with the plasma membrane to exert rapid and specific physicochemical effects (Buttgereit et al, 1999; Lipworth, 2000; Rhen et al, 2003; Shivaji & Jagannadham, 1992). These effects can alter membrane fluidity, vesicular fusion (Shivaji & Jagannadham, 1992) and ionic flux (Buttgereit et al, 1999), which are important for resistance to and repair of membrane injury. Recently, membrane stabilizing compounds such as poloxamer 188 (Spurney et al, 2011; Townsend et al, 2010), Mitsugumin 53 (Burkin & Wuebbles, 2012; Weisleder et al, 2012) and cromolyn sodium (Granchelli et al, 1996; Marques et al, 2008) have shown improvements to pathology, myofibre tension and cardiopulmonary function in dystrophin-deficient mice and dogs. The effects of glucocorticoids on membrane stability, however, have not been reported.

Because glucocorticoids act through multiple mechanisms, it has been unclear and controversial which molecular pathways provide efficacy in DMD and which are simply responsible for detrimental effects. For example, impaired growth is a glucocorticoid side effect for children with asthma (Avioli, 1993; Wolthers & Pedersen, 1990), but has been proposed as a pathway of efficacy in DMD by limiting muscle workload and delaying muscle maturation (Grounds & Shavlakadze, 2011). Further, immunotoxic effects contribute to reduced chronic inflammation, but recent evidence suggests ant-inflammatory NF-κB inhibition may be sufficient for efficacy (Peterson et al, 2011). It is clear, however, that detrimental effects of glucocorticoids currently limit their application; in DMD neonatal screening is not performed, and glucocorticoid regimens are delayed years until after the onset of fairly advanced symptoms. In other forms of muscular dystrophy, glucocorticoids are avoided altogether because the net balance of positive and negative effects is unclear. By investigating the molecular mechanisms of glucocorticoids, we have developed VBP15 as a novel oral drug. This compound is optimized for NF-κB inhibition, membrane insertion and glucocorticoid receptor (GR) specificity. Medicinal chemistry, however, both eliminates key glucocorticoid pathways and provides novel properties. Here, we present the discovery and mechanisms of this drug, then extensively examine efficacy and side effects in *mdx* muscular dystrophy model mice. We find VBP15 has novel membrane-stabilizing and immunological properties, and shows potent NF-κB inhibition and substantially reduced hormonal effects. To capitalize on this mechanism profile, which targets multiple pre-symptomatic defects, we adopt a prophylactic regimen, beginning dosing before *mdx* symptom onset in a blinded pre-clinical trial. This strategy would be analogous to a neonatal screening, preventive regimen in the clinic. Another intervention experiment in post-onset adult *mdx* mice shows repeatable efficacy in a different stage of disease. We find dose–response improvements with successful ablation of growth, bone and immunological toxicities seen with traditional glucocorticoids. These data provide new insights into biological mechanisms of efficacy *versus* side effects in DMD, identify VBP15 as a novel entity that warrants clinical investigation for DMD, and show therapeutic potential for other disorders of chronic inflammation and membrane instability.

## RESULTS

### *In vitro* characterization of VBP15

**Figure 1:**
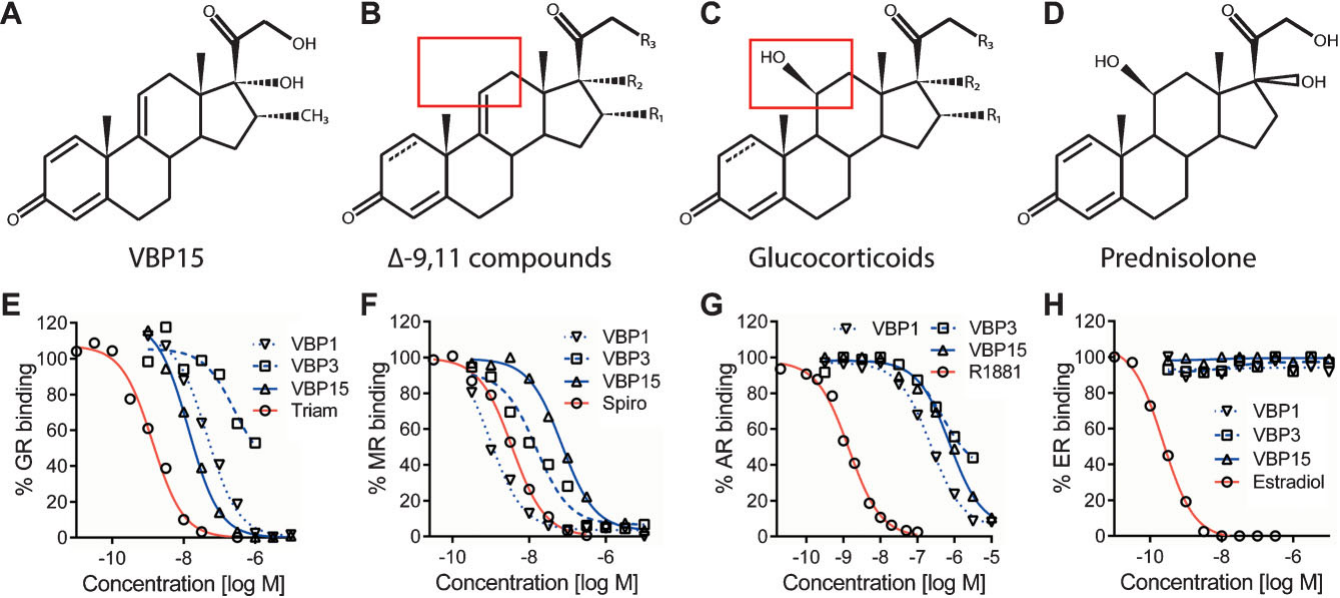
VBP15 structure differentiates it from glucocorticoids and improves GR specificity **A–D.** The chemical structure of VBP15 is provided (A). VBP15 was selected for clinical development by having the optimal profile from a library of Δ-9,11 compounds, whose general structure is provided in (B). Compound diversity for this library was generated by medicinal chemistry probing of the R_1_–R_3_ groups. These compounds are structurally related to glucocorticoids (C), but contain an essential Δ-9,11 double bond modification to the steroid C-ring, at the location depicted by the red box. This modification produces novel properties and clear differences in sub-activity profiles of these compounds. Prednisolone (D) is the active form of prednisone, a current glucocorticoid standard of care for DMD.**E–H.** Receptor specificity was determined through competitive binding assays. Here, radiolabeled high-affinity ligands were incubated with extracted steroid receptors and increasing concentrations of unlabeled competitor (high-affinity control, VBP15, VBP1 or VBP3). Best-fit curves are provided. VBP15 showed increased GR specificity through increased binding to the (E) GR and decreased binding to the (F) MR in comparison to other Δ-9,11 compounds (VBP1 and VBP3). VBP15 also showed low (H) androgen receptor binding and no detectable binding to the (G) oestrogen receptor. (Triam, triamcinolone; Spiro, spironolactone; R1881, methyltrienolone; Estradiol, 17β-estradiol).

VBP15 was selected as our lead compound for clinical development from a screening program focused on Δ-9,11 compounds. This Δ-9,11 class is differentiated from glucocorticoids by the key conversion of a hydroxyl group to a carbon-carbon double bond (Fig 1). Preliminary studies suggested these drugs had potential anti-inflammatory effects (Baudy et al, 2012) but lacked activation of a synthetic GR reporter. Through extensive medicinal chemistry probing the R_1_–R_3_ groups of the D-ring in this steroidal structure to generate a compound library, followed by multiple lines of screening studies focused on 20 candidates, VBP15 was subsequently identified as our lead compound. Selection was based upon its superior profile in an *in vitro* assay for NF-κB inhibition in myogenic cells, in addition to ligand-induced nuclear translocation of the GR, cytotoxicity, metabolite and pharmacokinetic properties (Reeves et al, 2013). To further screen candidate compounds for target receptor specificity, we performed competitive nuclear hormone receptor binding assays (Fig 1E–H). In these assays, we found that VBP15 shows increased specificity for GR binding in comparison to other Δ-9,11 compounds. For example, VBP15 exhibited an approximately 50-fold greater affinity for the GR than VBP3, and a 64-fold lower affinity for the mineralocorticoid receptor (MR). VBP15 also showed only very low affinity for the androgen receptor (Fig 1G), over 500-fold lower than the control methyltrienolone, and lacked any detectable binding to the oestrogen (Fig 1H) or progesterone (data not shown) receptors in these *in vitro* assays. From these screening, biochemical and specificity data, VBP15 presented a superior profile for therapeutic development.

**Figure 2:**
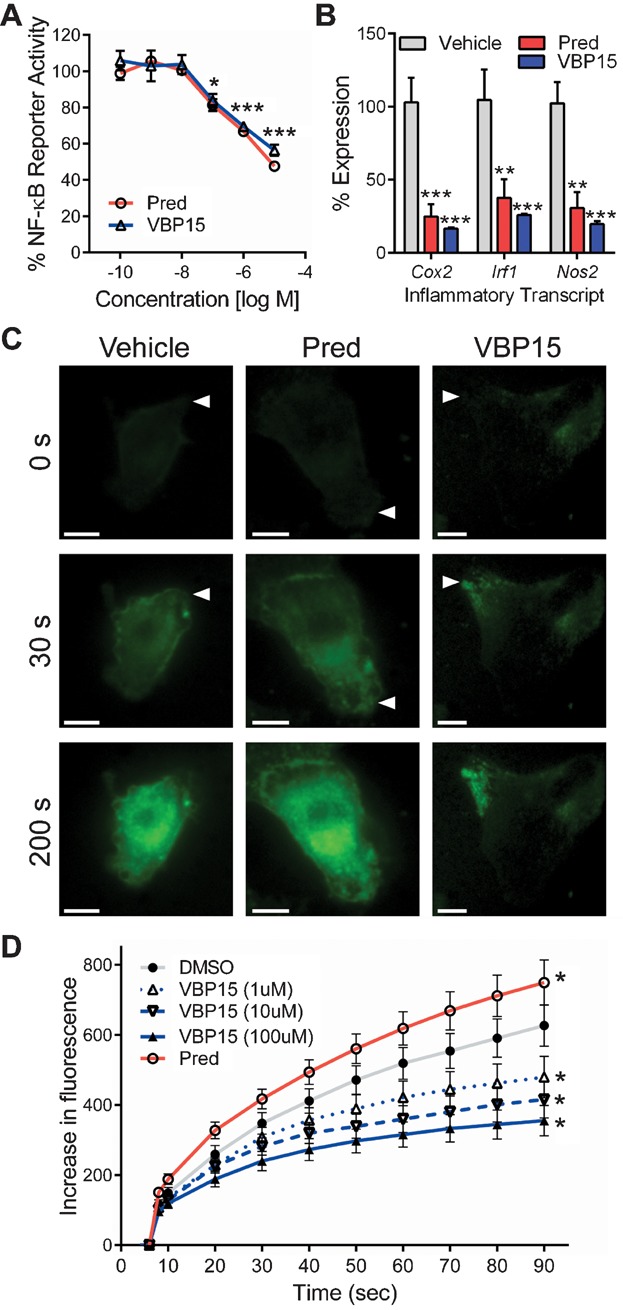
VBP15 inhibits inflammatory signaling and promotes membrane stability **A.** In an NF-κB reporter assay, increasing concentrations of prednisolone or VBP15 were applied to TNFα-induced C2C12 myoblasts stably expressing a luciferase reporter under the control of an NF-κB driven promoter. Significant reporter inhibition was observed at all concentrations over 10 nM.**B.** Inhibition was also observed for endogenous NF-κB activated inflammatory transcripts. *Cox2*, *Irf1* and *Nos2* expression was significantly reduced in TNFα-induced H2K myotubes, as determined by real time qPCR. Results are mean ± SEM from representative experiments performed in triplicate (ANOVA, *p < 0.05, **p < 0.005, ***p < 0.0005).**C,D.** Laser injury and dye exclusion assays to determine effects of VBP15 and prednisolone on cell membrane integrity. (C) Images of C2C12 myoblasts exposed to the indicated drug 15 min prior to laser wounding, with fluorescent visualization of FM1-43 dye entry into cells (white arrowheads mark sites of injury, scale bars = 5 µm). VBP15 shows protection against laser-induced injury. (D) Quantitation of FM1-43 influx over time (laser injury at time 6 s). VBP15 shows reduced impact of initial injury and enhanced repair, whereas prednisolone shows greater impact from injury with elevated dye uptake. Representative data from one of four experiments are presented as mean ± SEM. (ANOVA, *n* ≥ 16 per treatment, *p < 0.05; Pred, prednisolone).

Our studies here are benchmarked against prednisolone, the active form of prednisone. Both VBP15 and prednisolone inhibited TNFα-induced pro-inflammatory NF-κB signaling at similar levels in NF-κB reporter assays in C2C12 muscle cells at 1 nM or more (Fig 2A). To confirm effects on NF-κB target genes, several inflammatory transcripts known to be induced by TNFα were assayed by qPCR in VBP15- and prednisolone-treated H2K myotubes. We found VBP15 inhibited the TNFα-induced inflammatory transcripts *Cox2*, *Irf1* and *Nos2* (*p* < 0.005) at potencies similar to prednisolone (Fig 2B).

Both prednisolone and VBP15 are hydrophobic compounds that are expected to have physicochemical effects on lipid bilayers. We compared the effects of VBP15 and prednisolone on membrane injury and repair in live cells using an established laser injury assay (Sharma et al, 2012). Skeletal muscle cells treated with VBP15 showed reduced impact of the injury and enhanced repair in a dose dependent fashion (Fig 2C and D). Cells treated with prednisolone, however, showed greater impact from injury with elevated dye uptake. In this live single-cell injury model, prednisolone exacerbated, while VBP15 protected, injury to the plasma membrane.

### GR mediates VBP15 anti-inflammatory effects without inducing classical steroid transactivation

**Figure 3:**
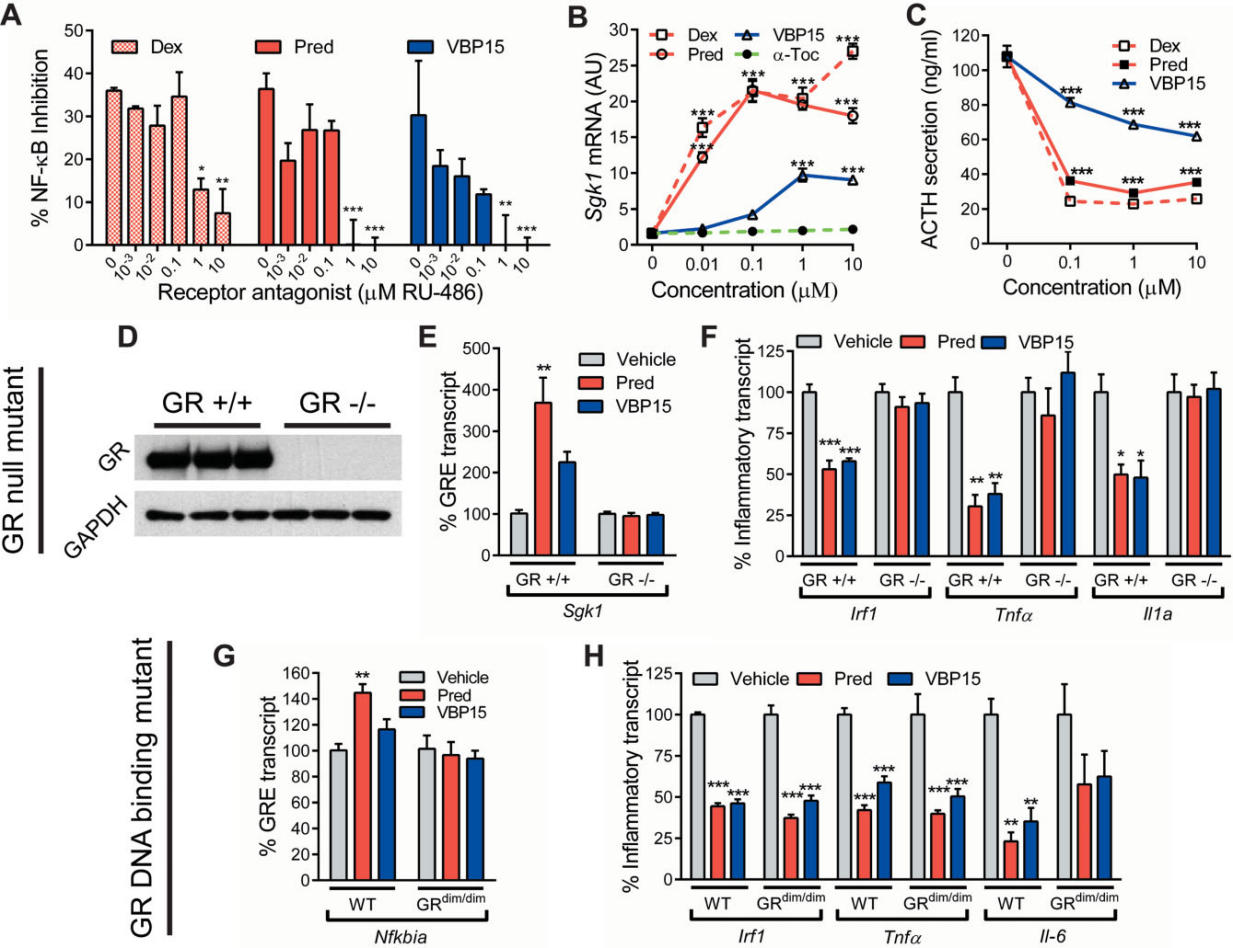
GR is required for anti-inflammatory activities of VBP15 and prednisolone, but VBP15 shows loss of GRE-mediated sub-activities associated with side effects **A.** Application of a steroidal receptor antagonist (RU-486) ablated the NF-κB inhibitory activity of both prednisolone and VBP15, indicating that both drugs share steroidal anti-inflammatory pathways.**B,C.** Assessment of GRE-mediated transcriptional (hormonal) activities differentiates prednisolone and VBP15. (B) *Sgk1* gene expression is controlled by a positive GRE and showed reduced activation by VBP15 in comparison to glucocorticoids in AtT-20 pituitary cells, as measured by qRT-PCR. α-Tocopherol was included as a negative control compound that lacks any GRE activity. (C) ACTH expression is controlled by a negative GRE, and is considered a component of adrenal suppression (negative side effect of pharmacological glucocorticoids). VBP15 showed reduced effects on this side effect pathway via ELISA of treated AtT-20 cell media.**D.** Western blot of *GR*^*null*^ fibroblasts and the control GR positive L929 line they were derived from, illustrating the absence of detectable GR protein in the *GR*^*null*^ cells. GAPDH was included as a loading control.**E,F.** *GR*^*null*^ and GR positive fibroblasts were treated with drug, then induced with TNFα and transcript levels assayed by real time qPCR. (E) *Sgk1* levels illustrate an absence of induction in *GR*^*null*^ cells, confirming *Sgk1* dependence on the GR and the absence of GR function in this cell line. (F) Inhibition of the endogenous NF-κB activated inflammatory transcripts *Irf1*, *Tnfα* and *Il1a* was observed in GR positive cells but not in *GR*^*null*^ cells, indicating the GR is essential for this inhibition.**G,H.** Spleens were harvested from *GR*^*dim/dim*^ and wild type control mice. Splenocyte suspensions were treated with drug, then induced with TNFα and transcripts assayed by qPCR. (G) *Nfkbia* levels illustrate an absence of GRE induction by the mutant GR, as well as a lack of induction of NF-κB inhibiting gene products, in *GR*^*dim/dim*^ splenocytes. (H) Inhibition of *Irf1*, *Tnfα* and *Il6* inflammatory transcripts was observed in both wild type and *GR*^*dim/dim*^ mutant splenocytes. This indicates *GR*^*dim*^ isoforms, which maintain protein-protein interactions but lose receptor-DNA interactions, still maintain inhibition of endogenous NF-κB activated inflammatory transcripts. (Pred, prednisolone; Dex, dexamethasone; α-Toc, vitamin E; ANOVA, *p < 0.05, **p < 0.005, ***p < 0.0005).

To investigate whether NF-κB inhibition by VBP15 is mediated by the same pathways as glucocorticoids, we examined the effects of the steroidal receptor antagonist, RU-486, on NF-κB inhibition. Increasing concentrations of RU-486 from 1 nM to 10 µM ablated NF-κB inhibition by VBP15 in a dose dependent manner, similar to results seen with prednisolone and dexamethasone (Fig 3A). This shows that the anti-inflammatory effects of VBP15, prednisolone and dexamethasone are all mediated through shared steroidal pathways.

A sub-activity of pharmacologic glucocorticoids that is largely separable from NF-κB inhibitor activities is the translocation of ligand-GR complexes to the nucleus where they directly mediate transcriptional pathways via glucocorticoid response elements (GRE) (e.g. classical steroid receptor transactivation or hormonal properties). Both positive- and negative-acting GRE-mediated transcriptional regulation has been described, and both forms of hormonal activities are more often associated with glucocorticoid side effects rather than efficacy, with some of these mediated by the pituitary (Diamond et al, 1990; Drouin et al, 1993; Itani et al, 2002; Meijsing et al, 2009; Yoshiuchi et al, 1998). In AtT-20 pituitary cells, we examined genes regulated by positive and negative GREs. *Sgk1*, a key mediator of fibrosis, is activated by a positive GRE. Both prednisone and dexamethasone (0.1 µM) showed a greater than 13-fold induction of *Sgk1* gene transcription, whereas VBP15 showed no such GRE-mediated transcriptional activity at the same concentration (Fig 3B). At 1.0 and 10 µM, VBP15 began to show some evidence of *Sgk1* transcriptional induction, but to a lower degree than traditional glucocorticoids. Adrenocorticotropic hormone (ACTH), the stimulatory hormone in adrenal steroidogenesis, is negatively regulated by a ligand/GR-GRE interaction (Drouin et al, 1993). Treatment with dexamethasone or prednisolone reduced ACTH secretion in AtT-20 cells to approximately 20% of untreated at all concentrations tested (Fig 3C). VBP15 produced more modest, dose-dependent effects on ACTH. qPCR of *Pomc*, the ACTH precursor, confirmed effects were consistent with transcription (data not shown). Thus, VBP15 has greatly reduced effects on both positively and negatively GRE-regulated transcripts in comparison to glucocorticoids, and might be expected to show a more favourable side effect profile.

Several mechanisms have been hypothesized for the inhibition of NF-κB by glucocorticoids and the GR. These include GRE-driven transactivation of genes that inhibit NF-κB, direct protein–protein interactions through which the GR may act as a corepressor when bound to NF-κB, and the activation of alternative receptors such as the MR. To investigate the mechanism by which glucocorticoids, VBP15 and/or the activated GR inhibit NF-κB, we performed further experiments in GR mutant cells. First, we tested whether the lack of GR in *GR*^*null*^ mutant fibroblasts affects the ability of drugs to inhibit inflammatory transcripts, which are predominantly controlled by NF-κB. Absence of the GR in this spontaneous mutant line was previously selected for (Housley & Forsthoefel, 1989) and confirmed here through Western blot (Fig 3D). Cells were then treated with prednisolone or VBP15 and inflammatory transcripts were induced with TNFα. Ablation of GR transactivation functions in *GR*^*null*^ cells was confirmed through qPCR of *Sgk1* transcript levels (Fig 3E). Examining inflammatory transcripts, we found *Irf1* (*p* < 0.0001), *Tnfα* (*p* < 0.05) and *Il1a* (*p* < 0.05) expression to all be significantly elevated in induced *versus* non-induced cells. In GR positive cells, both VBP15 and prednisolone inhibited the induction of *Irf1* (*p* < 0.005), *Tnfα* (*p* < 0.01) and *Il1a* (*p* < 0.05) to levels that were 30–58% of vehicle (Fig. 3F). In *GR*^*null*^ cells, neither drug was able to inhibit the induction of any of these transcripts. This data confirms that ligand-activated GR is essential for the inhibition of predominantly NF-κB driven inflammatory transcripts by both prednisolone and VBP15.

Next, primary splenocytes were harvested from control and *GR*^*dim*^ mutant mice. These mice contain a mutation in the DNA binding domain of the GR (Dahlman-Wright et al, 1991; Reichardt et al, 1998). This mutation prevents the GR from binding to DNA and activating dimer-driven GRE gene transcription, but maintains GR ligand-binding and protein–protein interactions. Here, primary splenocytes were treated with drug and induced with TNFα, then assayed by qPCR. First, we examined the induction of NF-κB inhibitor alpha (*Nfkbia*, or *IκBα*), a GRE-activated gene that also encodes an endogenous inhibitor of NF-κB. In wild type control splenocytes, *Nfkbia* expression was significantly increased by prednisolone (increase of 45 ± 13%, *p* < 0.005) but not by VBP15 (increase of 16 ± 16%) in comparison to vehicle. No induction was present with either drug in *GR*^*dim*^ splenocytes, demonstrating both the absence of GR dimer-driven gene expression in GR dim cells and a lack of induction of NF-κB inhibitory genes. Examining inflammatory transcripts, we found *Irf1* (*p* < 0.0001), *Tnfα* (*p* < 0.0001) and *Il6* (*p* < 0.05) were significantly elevated within induced *versus* non-induced primary splenocytes. Consistent with GR positive fibroblasts and H2K myotubes, treatment of wild type splenocytes with both VBP15 and prednisolone successfully inhibited the induction of all three inflammatory transcripts (*p* < 0.001) to levels that were roughly half those of vehicle. In contrast to *GR*^*null*^ genotype and GRE transcript experiments, we found that inhibition of all three inflammatory transcripts was maintained in the *GR*^*dim*^ mutant cells. Together, these experiments show that both prednisolone and VBP15 activate the GR to efficiently inhibit inflammatory transcription programs through protein–protein interactions, independent of DNA binding or transactivation of inhibitory genes.

### VBP15 improves dystrophic phenotypes in mice treated before the onset of early necrosis

The *mdx* mouse model of DMD shows staged histopathology, with little evidence of dystrophy from 0 to 3 weeks of age, then wide-spread necrosis from 3 to 6 weeks, followed by successful regeneration and a milder, more stable histological picture. We tested efficacy of VBP15 in the *mdx* model with treatment beginning prior to the 3 weeks onset of widespread pathology (prophylactic strategy). We carried out a blinded pre-clinical trial of pre-symptomatic mice with VBP15 (5, 15 or 30 mg/kg), prednisolone (5 mg/kg), or vehicle beginning at postnatal day 15 (PND15), following guidelines for robust pre-clinical trials and international SOPs (Landis et al, 2012; Nagaraju & Willmann, 2009; Spurney et al, 2009). These doses were chosen on the basis of favourable bioavailability, ADME and metabolite profiles (Reeves et al, 2013), as well as early safety studies in wild type mice by independent groups, which suggest the 28 day no-observable adverse effect level (NOAEL) of daily oral VBP15 in mice is at least 100 mg/kg. This dose range was chosen to better define the therapeutic window within this mouse disease model. The prednisolone dose was chosen based on our extensive pre-clinical experience with this drug in the *mdx* mouse model.

**Figure 4:**
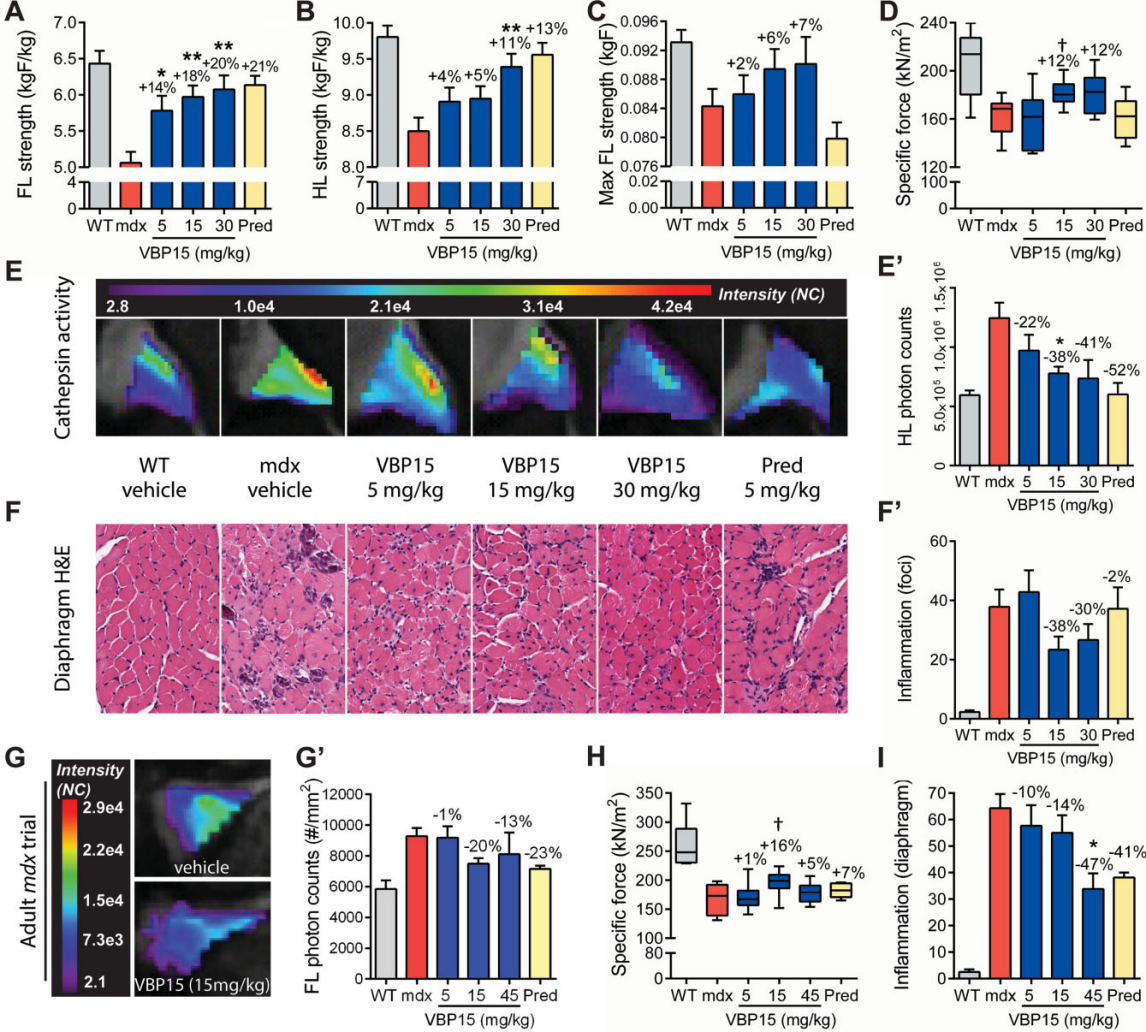
VBP15 improves dystrophic phenotypes of *mdx* mice in two pre-clinical trials (pre-symptomatic and post-onset treatment regimens) **A–F.** Prophylactic treatment of *mdx* mice beginning at 2 weeks of age showed dose-dependent improvement of clinical and histological endpoints. Mouse limb strength increased upon VBP15 treatment as measured by grip strength of 6 week old mice for both (A) forelimb and (B) hindlimb (*n* ≥ 12 mice/group). (C) Maximal force exerted by mouse forelimbs increased with VBP15 treatment but decreased with prednisolone treatment, due to prednisolone effects on mouse size (presented later). (D) Specific force of isolated EDL muscle increased with VBP15 treatment (*n* = 10 mice/group). (E) Live-animal imaging of cathepsin protease activity (ProSense680) shows reduced inflammation and necrosis of the hindlimbs in VBP15-treated *mdx* mice (E images; E′ quantitation of fluorescence; *n* ≥ 6 mice/group). (F) Histology of diaphragm muscle shows a decrease in inflammatory foci from VBP15 treatment at 15 and 30 mg/kg (F representative images, F′ quantitation; *n* = 6 mice/group).**G–I.** A second pre-clinical trial was performed in exercised adult *mdx* mice to assay post-onset efficacy. (G) Live-animal imaging of inflammation (ProSense680) showed a significant decrease with VBP15 treatment (G representative images, (G′) quantitation; *n* ≥ 6 mice/group). (H) Specific force of isolated EDL muscle was measured *ex vivo* at trial conclusion with 15 mg/kg VBP15 showing an increase consistent with the neonate trial (*n* ≥ 7 mice/group). (I) Histology of adult diaphragm showed a significant reduction in inflammatory foci upon 45 mg/kg VBP15 treatment (*n* = 6 mice/group). Values are mean ± SEM. For treatments, the mean percentage of increase or decrease of *mdx* vehicle values towards WT is provided. (Pred, prednisolone; FL, forelimb; HL, hindlimb; data exceeding 2 SD's was removed from specific force values as an outlier but included in all statistical analyses; one-tailed *t*-test of single dose *versus* vehicle *mdx* ^†^*p* < 0.05; ANOVA of dose-dependence groups *versus* vehicle *mdx* *p < 0.05, **p < 0.005, ***p < 0.0005).

Both VBP15 and prednisolone increased *mdx* forelimb and hindlimb normalized grip strength in comparison to vehicle (Fig 4A and B). Significant increases in VBP15 groups followed a dose-dependent pattern from 14% at 5 mg/kg (*p* < 0.05) to 20% at 30 mg/kg (*p* < 0.0005). For maximal force exerted, we again saw a dose dependent increase in forelimb strength upon VBP15 treatment, while prednisolone actually showed a reduction in maximal forelimb strength (Fig 4C). The discrepancy between prednisolone's effects on maximal and normalized force measures was due to the marked retardation of mouse growth induced by prednisolone, but not by VBP15 (see below). This indicates VBP15 increases functional mouse limb strength.

Evaluating muscle strength of isolated muscles *ex vivo*, extensor digitorum longus (EDL) muscles showed a reduction in specific force for *mdx* compared to WT (Fig 4D). While prednisolone showed no increase, specific force increased with VBP15 at both 15 and 30 mg/kg by an average of 12%. Following lengthening contractions, smaller drops in force for *mdx* EDLs after 10 contractions were observed for mice treated with prednisolone (7%) and VBP15 (11% at 15 mg/kg, *p* < 0.05), in comparison to vehicle (Supporting Information Fig 1A). These data suggest functional benefits to isolated dystrophic muscles.

Optical imaging of live animals was used to monitor muscle inflammation. ProSense 680, a substrate cleaved by cathepsin proteases upregulated in DMD (Kar & Pearson, 1978; Takeda et al, 1992), was injected as previously reported (Baudy et al, 2011). Cathepsin activity was elevated in *mdx* mice (Fig 4E, Supporting Information Fig 1B and C). VBP15 and prednisolone decreased cathepsin activity towards WT levels. Decreases in VBP15 groups followed a dose-dependent pattern, from a 22% decrease in comparison to vehicle at 5 mg/kg to a 41% decrease at 30 mg/kg in hindlimbs. This suggests VBP15 reduces muscle inflammatory disease *in vivo*.

In histopathology studies, quantitative H&E analysis of *mdx* diaphragms revealed a clear inflammatory phenotype, with 16-fold higher inflammatory cell counts compared to WT (Fig 4F). Mice treated with VBP15 at 15 and 30 mg/kg displayed 38 and 30% reductions in inflammatory foci compared to vehicle. VBP15 also reduced calcified fibres (Supporting Information Fig 1D). These data are evidence that VBP15 reduces inflammation and improves inflammatory muscle pathology.

### VBP15 improves dystrophic phenotypes in adult *mdx* mice treated after symptom onset

In a separate trial, exercised adult *mdx* mice were treated for 4 months. In agreement with the pre-symptomatic trial above, ProSense680 live animal imaging exhibited a 20% and 13% decrease in muscle inflammation upon VBP15 treatment at 15 and 45 mg/kg (Fig 4G). Isolated EDLs showed a 16% increase in specific force upon treatment with VBP15 at 15 mg/kg (Fig 4H). H&E histology revealed VBP15 significantly decreased diaphragm inflammation (Fig 4I). These data reinforce VBP15 efficacy and indicate both pre-symptomatic and post-onset treatment regimens can benefit disease.

### VBP15 does not display immunotoxicity seen with prednisolone

**Figure 5:**
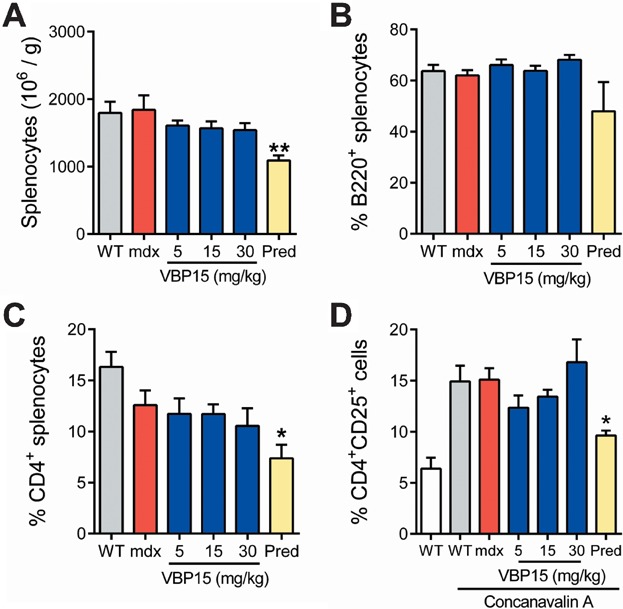
VBP15 does not show immunosuppressive activities shown by prednisolone Prednisolone significantly reduced the number of viable splenocytes per gram of spleen tissue, whereas VBP15 did not at any dose.The percentage of B lymphocytes, as measured by FACS analysis of B220 positive cells, was reduced in spleens from prednisolone treated *mdx* mice, while VBP15 showed no decrease in B cells.Spleen CD4^+^ T cell numbers were significantly decreased in prednisolone treated *mdx* spleens, but not by VBP15 treatment.Activation of *mdx* splenocyte T cells by concavalin A was impaired by prednisolone, but not impaired by VBP15 treatment. Values are mean ± SEM. (Pred, prednisolone; (A) *n* ≥ 12, (B–D) *n* = 3–5; *p ≤ 0.05, **p < 0.005).

Pharmacologic glucocorticoids show immunosuppressive and immunotoxic properties that limit therapeutic windows and long-term prescription. We benchmarked VBP15 against prednisolone to determine if similar sub-activities were seen. Untreated *mdx* mice showed enlarged spleens and increased numbers of peripheral blood leucocytes (PBLs) compared to WT mice (Supporting Information Fig 2A and B). VBP15 treatment reduced spleen mass and PBL counts in a dose-dependent manner to levels resembling WT. Prednisolone reduced these measures below WT, suggesting immunosuppressive and/or immunotoxic properties. Further, prednisolone significantly decreased viable splenocytes per gram of tissue (*p* < 0.005), while this was not observed for any VBP15 dose (Fig 5A).

We next examined effects of VBP15 on B and T lymphocytes isolated from *mdx* spleens at the trial conclusion. Both B lymphocytes and CD4^+^ T lymphocytes were depleted by prednisolone but not VBP15, as measured by percent B220^+^ and CD4^+^ positive splenocytes, respectively (Fig 5B and C). CD4^+^ T cell activation was assayed by stimulation of splenocytes with concanavalin A (ConA). Prednisolone treatment significantly reduced activated CD4^+^CD25^+^ cells (*p* = 0.01), while VBP15 did not. Taken together, these findings suggest VBP15 modulates inflamed *mdx* immune systems towards a WT state, while prednisolone treatment leads towards an immunocompromised state.

### VBP15 shows a superior side effect profile compared to pharmacological glucocorticoids

**Figure 6:**
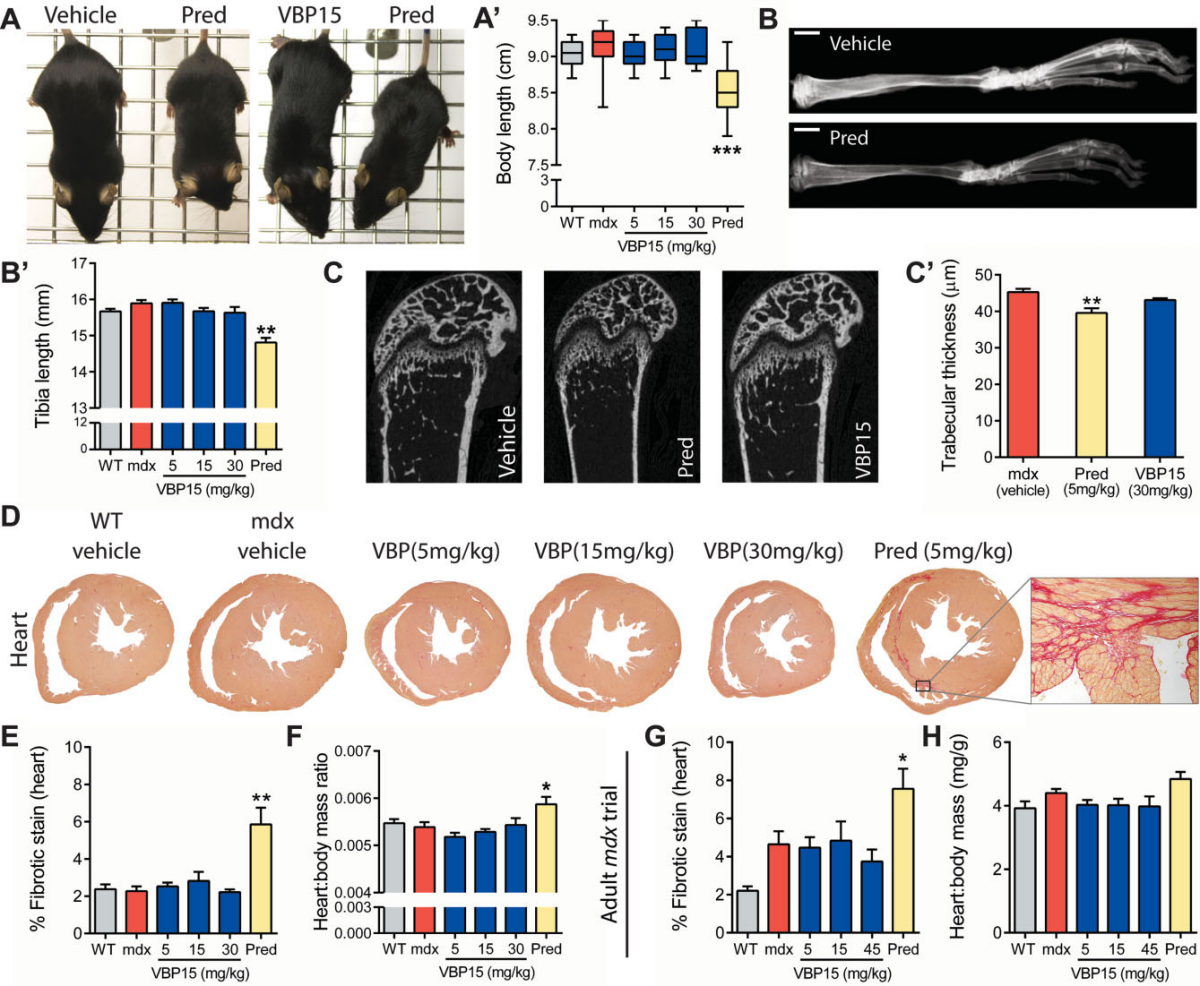
VBP15 lacks the side effects of current glucocorticoid regimens *in vivo* **A.** Prednisolone treatment stunted the growth of developing mice in comparison to both vehicle and VBP15 groups. Representative photographs (A) and quantitation of body length (A′) are provided.**B.** Bone lengths were reduced upon prednisolone treatment. X-rays (B) of mouse tibias illustrate size differences (scale bars = 2 mm). Quantitation shows a significant decrease in tibia length (B′).**C.** MicroCT imaging analysis of femur revealed a significant decrease in trabecular thickness (C′) for prednisolone treated mice.**D–F.** Increases in cardiac fibrosis and heart mass were detected in prednisolone treated mice, suggestive of cardiac damage as a side effect lacking for VBP15. Sirius red staining of cardiac muscle shows increased fibrosis in prednisolone-treated mice, but not VBP15 mice. Representative images (D) and digital quantitation of fibrosis (E) are provided. To the right of the image panel is a higher magnification image from the area outlined in box. (F) Heart mass ratios were increased by prednisolone but not by VBP15.**G,H.** In adult *mdx* mice as well, increases in cardiac fibrosis (G) and heart mass (G) were observed with prednisolone treatment but not VBP15 treatment. In adult *mdx* vehicle mice, an expected disease- and age-related increase in fibrosis over WT is seen. Values are mean ± SEM. (*n* ≥ 12 per group for (A,B,E,F); *n* ≥ 5 for (C,G,H); *p < 0.05, **p < 0.005, ***p < 0.0005).

Stunted growth is a significant side effect of chronic prednisone use in children (Avioli, 1993; Wolthers & Pedersen, 1990). In our pre-symptomatic *mdx* study, prednisolone treatment significantly stunted the growth of young mice (Fig 6A). After 5 weeks of treatment, *mdx* mice receiving prednisolone were significantly shorter (8.6 ± 0.4 cm) than vehicle (9.1 ± 0.3 cm, *p* < 0.001). No significant reduction in body length was observed for any VBP15 dose.

Chronic treatment with glucocorticoids negatively affects bone growth and development, and can cause osteoporosis (Bircan et al, 1997; Manolagas & Weinstein, 1999). Tibia length was measured to determine if VBP15 inhibited bone growth (Fig 6B). Vehicle *mdx* mice had tibia lengths of 15.9 ± 0.3 mm, while prednisolone significantly decreased this to 14.8 ± 0.5 mm (*p* < 0.005). VBP15, however, did not affect tibia length at any concentration. MicroCT was performed on femurs to examine bone density and structure (Fig 6C). Comparison of vehicle, prednisolone and the highest VBP15 dose showed prednisolone to significantly reduce trabecular thickness (*p* < 0.005) compared to vehicle, while VBP15 did not. Prednisolone thus demonstrated side effects to bone not observed with VBP15 treatment.

We have previously reported deleterious effects of prednisone on increased fibrosis in *mdx* hearts (Sali et al, 2012). In both pre-clinical trials (pre-symptomatic and adult), we examined cardiac and skeletal muscle for measures of fibrosis. In the pre-symptomatic trial (Fig 6D–F), prednisolone caused a significant elevation of heart mass ratios over vehicle (5.9 ± 0.6 *vs*. 5.4 ± 0.4, *p* < 0.05), indicative of cardiac hypertrophy. No increase was present in VBP15 groups. Histologically, clear fibrosis was evident in 50% of young (8 weeks) prednisolone-treated hearts compared to 0% of all other groups. Histological analyses of skeletal muscle (gastrocnemius) also showed increased fibrosis in prednisolone-treated mice (8.1 ± 2.2%, *p* < 0.05) compared to vehicle-treated (4.2 ± 1.8%), VBP15-treated (3.5 ± 1.2% at 30 mg/kg), and WT (2.0 ± 0.5%) mice (Supporting Information Fig 2C–E). In the adult trial, cardiac findings were consistent with the pre-symptomatic trial (Fig 6G and H). Here as well, prednisolone treatment increased fibrosis and mass ratios of *mdx* hearts, while VBP15 did not.

## DISCUSSION

**Figure 7:**
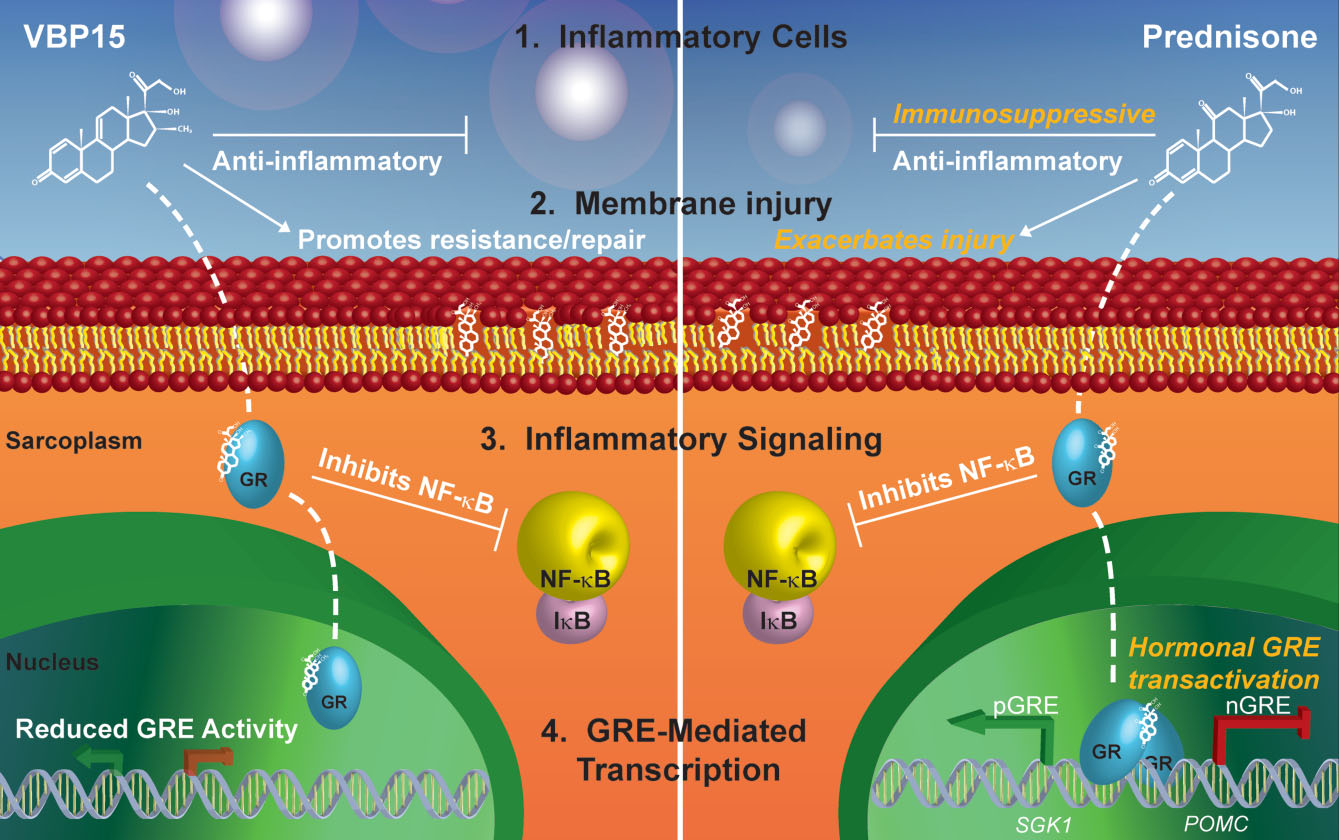
Working model of VBP15 and prednisone drug mechanism sub-activity profiles Steroidal compounds such as glucocorticoids (prednisone) and Δ-9,11 compounds (VBP15) are multi-potent drugs. Through dissecting the sub-activities of these compounds, we find that: (1) VBP15 reduces inflammation but does not show the immunosuppressive impairment of lymphocyte viability and function observed for prednisone. (2) Within an environment of plasma membrane disruption, VBP15 helps to promote resistance to and repair of injuries, while prednisone can exacerbate membrane injury. (3) Inside cells, both compounds bind to and activate the GR to potently inhibit inflammatory NF-κB signaling through protein–protein interactions. (4) Though they both bind to the GR, prednisone causes strong induction of hormonal GRE controlled promoter elements, while VBP15 eliminates or greatly reduces these effects.

Development of mechanisms to improve muscular dystrophy in the absence of detrimental hormonal effects will substantially improve DMD patient medical care, could provide a therapy for dystrophies with no current treatment, and could improve care of diverse chronic inflammatory disorders. Here, we describe the development, mechanisms and effects of a novel drug that dissects and optimizes several sub-activities of classic glucocorticoids (Fig 7), demonstrating it is possible to treat muscular dystrophy in the absence of growth, hormonal and immunosuppressive side effects. For one sub-activity, we show VBP15 has protective physicochemical effects on the plasma membrane, protecting cells from injury and promoting membrane repair. This sub-activity is likely to be particularly important in DMD where disease pathogenesis is clearly linked to membrane instability and myofibre injury. For another, we show that a key anti-inflammatory activity, inhibition of TNFα-induced NF-κB, is retained by VBP15. We further show that this mechanism occurs through protein–protein interactions of the VBP15 ligand-activated GR, independently of DNA binding, GRE activation, or upregulation of inhibitory transcripts. We have previously shown that NF-κB activation is among the earliest histological features of DMD neonates (Chen et al, 2005; Porter et al, 2002, 2003), years before symptoms appear. This, coupled with the results of our blinded *mdx* pre-clinical data here, suggests that very early treatment of DMD patients with VBP15 may prevent or delay the onset of some clinical symptoms. Finally, the well-documented and extensive side effect profiles of glucocorticoids, inclusive of immunotoxicity, growth stunting and effects on pituitary function, were not seen with VBP15 at doses up to nine times prednisolone dosing. These properties provide us with a new mechanistic profile with which to approach both patient therapy and scientific questions regarding inflammation, signaling and disease mechanisms.

Steroidal compounds are multi-mechanistic by nature and display physicochemical effects on the plasma membrane (Rhen et al, 2003; Shivaji & Jagannadham, 1992). We find VBP15 and prednisolone differ in their effects on membranes, with VBP15 treatment protecting live cells from laser-induced injury. Membrane-stabilization is a property that is analogous to poloxamer 188, Mitsugumin 53 or cromolyn sodium, which operate through varying mechanisms and show beneficial effects on dystrophin deficient dog and mouse muscle *in vivo* (Marques et al, 2008; Townsend et al, 2010; Weisleder et al, 2012). Membrane-stabilizing effects of VBP15, but not prednisolone, are consistent with increases in specific force observed for VBP15 but not for prednisolone. VBP15 effects on membrane stability could be explained by altered compression of phospholipid head groups within the membrane, altered ion balances (Howard et al, 2011), altered membrane or vesicular fusion (Shivaji & Jagannadham, 1992), or altered oxidative stress at the plasma membrane (Howard et al, 2011; Kavanagh & Kam, 2001; Marques et al, 2008; Saija et al, 2001). With membrane integrity and repair becoming of increasing importance in muscle (Bansal et al, 2003; Jaiswal et al, 2007), cardiovascular (Chase et al, 2009), neurodegenerative (Bazan et al, 2005) and airway (Gajic et al, 2003) disorders, physicochemical properties of VBP15 will be an intriguing area of investigation moving forward.

Chronic treatment with glucocorticoids (prednisone, deflazacort) is the current standard of care for DMD, yet glucocorticoids are well-known to induce muscle atrophy pathways via *FOXO1*, stunt the growth of paediatric patients, and can suppress the immune system which plays an important role in myofibre repair cycles. Thus, clinical improvements in DMD patients treated with glucocorticoids may be the sum balance of beneficial anti-inflammatory effects and deleterious pathways. Both *in vitro* and *in vivo* data presented here are consistent with this model. In *mdx* mice, we find the net balance of prednisolone treatment increases normalized strength, however at the same time it stunts the growth of mice resulting in lower maximal strength, is immunosuppressive, and increases the presence of muscle damage. We, as well as others in recent reports (Bauer et al, 2009), also find that prednisolone increases cardiac fibrosis in *mdx* mice. Comparable examination of cardiac fibrosis in glucocorticoid treated DMD patients has not been examined directly in the literature, however recent anecdotal cardiac MRI reports show substantial fibrosis, suggesting this may be an intriguing area of investigation moving forward, with a possibility to develop more “heart healthy” treatments. VBP15 however does not stunt the growth of mice, shows no evidence of splenocyte immunotoxicity, and does not increase muscle fibrosis in skeletal or cardiac muscle. In the absence of these side effects, VBP15 increases strength, increases absolute and specific force measures and decreases muscle inflammation. A comparison of VBP15 and prednisolone mechanistic profiles in the context of these results has several implications. One, stunted growth appears to be a side effect of glucocorticoid treatment in DMD as opposed to a mechanism of efficacy, as has been logically proposed in the past (Grounds & Shavlakadze, 2011) by limiting body size to reduce muscle workload. Here, we see dose-dependent increases in *mdx* strength in the absence of stunted growth, suggesting the potential to increase patient strength without overt effects to growth and development. Two, immunotoxicity is a potential side effect of glucocorticoid treatment – not the primary cause of efficacy. This is supported by the failure of general immunosuppression to increase DMD patient strength (Griggs et al, 1993; Kissel et al, 1993). Three, NF-κB inhibition is shared by both drugs, supporting our hypothesis that this is a shared pathway of efficacy. In further support of this, peptides or antibodies targeting NF-κB pathways benefit *mdx* phenotypes (Grounds & Torrisi, 2004; Peterson et al, 2011), while in contrast, constitutive activation of NF-κB causes severe muscle wasting (Cai et al, 2004; Mourkioti et al, 2006). Finally, GRE transactivation appears to be disposable to efficacy in DMD. As GRE-regulated genes have been implicated in a number of glucocorticoid side effects, this is particularly exciting because it provides a clear avenue by which to reduce harsh side effect profiles currently limiting the use of these widely applicable drugs. Indeed, along with a reduction in GRE activity for VBP15, we also see an absence of glucocorticoid side effects in *mdx* mice. Importantly, efficacy is maintained in the absence of immunosuppression and overt hormonal effects to growth and development, providing insight into mechanisms of glucocorticoids in muscular dystrophy and demonstrating a successful separation of pathways into dystrophy efficacy and side effects.

Exon skipping represents another promising line of therapeutic development for DMD (Hoffman et al, 2011). Short of gene replacement, this may offer the greatest potential to alleviate DMD because it aims to restore expression of disease-causing dystrophin deficiencies. However, isoforms induced by exon skipping, as well as the mini-gene dystrophin constructs envisioned in gene therapy, are also expressed in Becker muscular dystrophy (alleles of dystrophinopathy leading to milder disease). In other words, both exon skipping and gene therapy are expected to mitigate but not cure disease. We and others find exon skipping partially restores specific force deficits in *mdx* muscle, with *ex vivo* force contractions typically showing approximately 20% increases over *mdx* controls (Aoki et al, 2010; Dumonceaux et al, 2010). This likely represents an upper limit to therapeutic strength increases, short of gene replacement. We find VBP15 treatment increases EDL specific force as well, with 15 mg/kg producing 12 and 16% increases in the two trials presented here. Currently, patients with Becker or other milder muscle dystrophies are not routinely administered prednisone (Johnsen, 2001) due to the unclear net balance of detrimental *versus* beneficial effects it would provide. Evidence here suggests VBP15 could provide a novel therapy for Becker's and other milder dystrophies, or serve as a valuable combination therapy used with exon skipping to provide efficacy through independent mechanisms.

Currently, glucocorticoid regimens for DMD delay treatment to avoid serious detriment, and many patients eventually discontinue treatment as a result of side effects. A drug lacking such harsh effects has the potential to change physicians' treatment approaches since it would be more amenable to a chronic treatment regimen, and would enable treatment during pre-symptomatic or late stages when many patients are not taking prednisone. This rationale prompted us to change our approach to *mdx* preclinical trial design for VBP15, and indeed we saw clear efficacy with an ablation of side effects to *mdx* growth, bone and muscle. Intriguingly, by enabling treatment of DMD at pre-symptomatic ages, a strong rationale for neonatal screening could be built to move forward towards a preventative medicine approach to treatment, thereby improving the way we diagnose and treat DMD patients.

International consensus has established the *mdx* mouse as the model of choice for preclinical and proof-of-concept studies because they represent the exact monogenic biochemical defect present in DMD (Nagaraju & Willmann, 2009; Willmann et al, 2009, 2012). However, *mdx* mice present a milder disease than DMD, with peak severity from approximately 3–8 weeks of age after which they recover substantially until advanced ages. This prompts various strategies to exacerbate the *mdx* phenotype. One strategy is to introduce additional mutations which exacerbate disease onto the *mdx* background, examples of which include the *mdx:utrophin*^−/−^ (Deconinck et al, 1997; Grady et al, 1997b), *mdx:adbn*^−/−^ (Grady et al, 1999), *mdx: α7 integrin*^−/−^ (Guo et al, 2006), *mdx:PV*^−/−^ (Raymackers et al, 2003) and *mdx:MyoD*^−/−^ (Megeney et al, 1999) double knockout models. These provide advantages through increased disease severity and a differing array of symptoms, which allow for more efficient trials utilizing smaller sample sizes without the added need of forced exercise protocols. Several groups have thus utilized *mdx:utrophin*^−/−^ double transgenic mice to successfully detect therapeutic efficacy (Delfin et al, 2011; Gehrig et al, 2012; Goyenvalle et al, 2010; Wakefield et al, 2000). It is possible for a second mutation to introduce underlying biochemical or biological differences however, for example *utrophin*^−/−^ single transgenic mice have an increased susceptibility to seizures (Knuesel et al, 2002), along with altered neuromuscular junction folding and altered acetyl choline receptor density (Grady et al, 1997a), which could feasibly affect neuromuscular disease outside of a direct consequence of dystrophin deficiency (Willmann et al, 2009). Here, we chose to use larger sample sizes of monogenic *mdx* mice to ensure that the efficacy parameters we measured were from phenotypes directly resulting from dystrophin deficiency. To optimize our trial designs, we adopted two strategies to measure *mdx* phenotypes at points of increased severity, in one trial by assaying young mice during natural peaks in disease severity, and in the other by using forced exercise protocols in adult mice to exacerbate disease. Through both strategies, we consistently detect significant *mdx* phenotypes and VBP15 efficacy through an improvement of *mdx* phenotypes towards wild type.

Extension of VBP15 to other clinical disorders of membrane instability and chronic inflammation will require further studies and clinical development. Studies of VBP15 in animal models of arthritis, asthma, multiple sclerosis and inflammatory bowel diseases are currently underway. Through collaboration with the Muscular Dystrophy Association Venture Philanthropy, and the National Institutes of Health Therapeutics for Rare and Neglected Disease (TRND) program, VBP15 is being actively developed for DMD as the initial indication. Participation in these programs has provided repeatability, efficacy and safety through independent trials both *in vitro* and *in vivo*. VBP15 shows favourable pharmacokinetic, ADME and metabolite profiles (Reeves et al, 2013). Early safety studies by independent groups suggest a single dose tolerance of at least 500 mg/kg in mice, and a 28 day NOAEL of at least 100 mg/kg in mice, which is more than twice the highest doses (30 and 45 mg/kg) used here to show both efficacy and a clear reduction in side effects in comparison to prednisolone.

Our results demonstrate the successful separation of pathways providing efficacy from side effects in muscular dystrophy. The translation of these into model mice by treatment with a novel, orally available drug, indicates that strength and pathology phenotypes can be improved by treatment without overt hormonal, growth or immunosuppressive effects. VBP15 merits further investigation for efficacy in clinical DMD trials, and is relevant to a diverse group of disorders through shared inflammation or membrane injury molecular pathways. By focusing on DMD as an initial indication, we benefit from having (i) models reproducing the ubiquitous molecular deficit present in all patients, and (ii) a homogenous patient population with strong foundations providing national clinical trial support. Movement into the clinic would improve treatment of human disease, provide further mechanism insight and provide a template for future drug development. With a molecular profile relevant to diverse disorders and DMD amenable to neonatal screening, VBP15 may provide an excellent opportunity to develop an Orphan disease therapy in a way that helps larger groups of more complex disorders.

## MATERIALS AND METHODS

### NF-κB inhibition

C2C12 cells stably expressing an NF-κB luciferase reporter were cultured and assayed as described previously (Baudy et al, 2009). For GR antagonist experiments, cells were treated with a constant concentration of drug (1 µM) and increasing RU-486 (Sigma) concentrations. In both experiments, cells were pretreated with drug for 1 h, stimulated with TNFα (10 ng/ml) and assayed for luciferase activity 3 h later. H2K myoblasts were cultured with gamma-interferon at 33°C, and differentiated into myotubes in six-well plates with Matrigel at 37°C. Cells were plated 1E5 per well, treated with drug after 4 days of differentiation, induced with TNFα 24 h later, and RNA harvested the following day.

### Pituitary cell assays

For pituitary cell line experiments, AtT-20/D16v-F2 cells (ATCC) were maintained at 37°C, 5% humidity in DMEM with 10% FBS. In *Sgk1* studies, cells were plated at 6E5 per well overnight, then serum starved in six-well plates. After 48 h, cells were drug treated for 6 h then lysed for RNA. For ACTH studies, cells were plated at 1.3E6 per T25 flask and treated with drug. Media and drug were changed daily for 5 days, then cells were counted and replated in six-well plates. Twenty-four hours later, media was collected and cells lysed for RNA. ACTH secretion was assessed by lumELISA (Calbiotech).

### GR mutant assays

*GR*^*null*^ cells and the parental L929 fibroblast line they were derived from (Housley & Forsthoefel, 1989) were cultured in DMEM at 37°C. Protein lysates were obtained from untreated cells using RIPA buffer, separated on 4–15% PAGE gels, and transferred to nitrocellulose membranes, which were immunoblotted with rabbit polyclonal anti-GR (Santa Cruz) and rabbit monoclonal anti-GAPDH (Cell Signaling Technology), followed by HRP-secondary (Bio-Rad). For assays of GRE and inflammatory transcripts, cells were treated with drug for 24 h, then stimulated with TNFα (1 ng/ml) for an additional 24 h, lysed for RNA, and assayed by qPCR.

*GR*^*dim/dim*^ mice (Reichardt et al, 1998) were obtained from the Deutsches Krebsforschungszentrum (German Cancer Research Center). *GR*^*dim/dim*^ and wild type control (C57BL/6) mice were maintained in an animal facility within IACUC guidelines under approved protocols. Spleens were isolated and single cell suspensions generated through homogenization and lysis of red blood cells using ACK lysis buffer (Lonza). Splenocytes were treated with drug for 24 h, then stimulated with TNFα (10 ng/ml) for another 24 h. RNA was extracted from splenocytes, with analysis of GRE and inflammatory transcripts performed by qPCR.

### Real-time qPCR

cDNA was produced using the High Capacity cDNA Reverse Transcription Kit (ABI). Transcript levels were analysed via TaqMan qPCR assays (LifeTech). The following assays were used: *Sgk1*, Mm00441380_m1; *Pomc*, Mm00435874_m1; *Irf1*, Mm01288580_m1; *Cox2*, Mm03294838_g1; *Nos2*, Mm00440502_m1; *Tnfα*, Mm00443258_m1; *Il1a*, Mm00439620_m1; *Nfkbia*, Mm00477800_g1; *Il6*, Mm00446190_m1. qPCR was performed using TaqMan gene expression master mix and 18s rRNA as a normalization control (ABI).

### Laser-mediated wounding of live cells

C2C12 myoblasts were pretreated with drug in growth media for 15 min. Immediately following this, cells were wounded in imaging media (Hank's Balanced Salts, 10 mM HEPES, pH 7.4) containing drug or equivalent vehicle, 2 mM Ca^2+^ and 2 µg/ml FM1-43 dye (Molecular Probes Inc.) at 37°C. Injuries were performed with a pulsed one-photon laser (‘Ablate’, Intelligent Imaging Innovations Inc.) and a custom built Olympus IX81 microscope (Olympus America). Wounding was performed with ablation power 116 in a 2 × 2 µm^2^ for all injuries. Cells were imaged at 2 s intervals. Initial fluorescence intensity was measured and used to normalize subsequent time points. Fluorescence intensity over time was measured within cell borders using SlideBook 5.0 (Intelligent Imaging Innovations Inc.).

### Receptor binding assays

The various steroid receptors (GR, MR, ER, AR and PR) were extracted and incubated with a constant concentration of radiolabeled, high-affinity ligand. Increasing concentrations of unlabeled VBP1, VBP3, VBP15 or high-affinity ligand controls (triamcinolone, spironolactone, 17-β-Estradiol, methyltrienolone or Promegestone) were added and the percent binding of radiolabeled ligands determined to gauge the affinity of the unlabeled competitors for the steroid receptors.

### Animal care and drug dosing

Two separate *mdx* trials were performed to provide repeatability as well as contrasting treatment and phenotyping regimens. The larger “pre-symptomatic” *mdx* trial (78 mice total) is presented here as the primary trial. WT (C57BL/10ScSnJ) and *mdx* (C57BL/10ScSn-*Dmd*<*mdx*>/J) mice were obtained from Jackson Laboratory (Bar Harbor, ME). All experiments were conducted within IACUC guidelines under approved protocols. PND15 was chosen as the trial start point because it was the earliest age prednisolone could confidently be safely administered (Heine & Rowitch, 2009; Pinsky & Digeorge, 1965). At this point, mice were divided into groups of equally matched body mass, which were then blinded to both drug and genotype for subsequent phenotyping and histology experiments. Treatment groups (*n* = 12–14 per group) consisted of WT vehicle, *mdx* vehicle, *mdx* VBP15 (5, 15 or 30 mg/kg), and prednisolone (5 mg/kg). Mice received daily AM dosing via cherry syrup vehicle at 1 µl per 1 g body weight. One mouse suffered a head injury during phenotyping and was removed from subsequent experiments. No adverse effects from drug treatment were observed. Functional phenotyping was performed in 5-week-old mice. *In vivo* imaging was performed in 6–7 week old mice. At 8 weeks of age, terminal assays were performed and tissues harvested.

A separate ‘adult’ *mdx* trial (48 mice total) was performed during lead compound identification according to established standard operating procedures. In this smaller, open-label study, WT and *mdx* mice (*n* = 8 per group) received daily PM oral syrup vehicle, prednisolone (5 mg/kg) or VBP15 (5, 15 or 45 mg/kg). All mice were subjected to 30-min run on horizontal treadmills at 12 m/min, twice a week except during data collection to unmask the mild phenotype of *mdx* mice. One death was recorded at VBP15 45 mg/kg body weight. Mice were administered drug for 4 months starting at 6 weeks of age.

### Motor function

At 5 weeks of age, mice in the neonate trial were assayed for motor function via grip strength measurement. Strength was assessed daily AM for 5 days using a grip strength meter (Columbus Instruments). Data was interpreted as maximum daily values for each of five testing days and averaged over the 5 days. Animals were acclimated for 1 week prior to data collection.

### Live imaging

Mice were anaesthetized with isoflurane, and cathepsin caged near-infrared imaging was performed on 6–8 mice per group as described previously (Baudy et al, 2011). Briefly, mice received intraperitoneal (IP) injections of ProSense 680 (Perkin–Elmer) in PBS 24 h prior to imaging within an Optix MX2 Imager (ART). Scans of uninjected mice were performed to obtain baseline optical intensity measurements. Forelimb and hindlimb measurements were made at 0.5 mm resolution and analysed using Optiview software.

### *Ex vivo* force contractions

At trial endpoint, EDL muscle was isolated from live anaesthetized mice and placed in Ringer's solution (137 mM NaCl, 24 mM NaHCO_3_, 1 mM glucose, 5 mM KCl, 2 mM CaCl_2_, 1 mM MgSO_4_, 1 mM NaH_2_PO_4_ and 0.025 mM tubocurarine chloride) at 25°C bubbled with 95% O_2_ and 5% CO_2_. Contractile properties were measured *ex vivo* according to established methods (Brooks & Faulkner, 1988) using a force apparatus (model 305B, Aurora Scientific). Drop in force was measured after 10 lengthening contractions where each muscle was stretched over 10% of its length.

### Immunotoxicity studies

Peripheral blood was obtained via retro-orbital bleed. Following sacrifice, spleens and thymuses were harvested, weighed, and processed to generate single cell suspensions of splenocytes and thymocytes, respectively. Red blood cells in splenocytes and peripheral blood were lysed with 3% acetic acid + methylene blue (Stem Cell Technologies). All leucocytes were quantified via haemocytometer. For lympho-phenotyping studies, splenocytes were stained for FACS with FITC-conjugated anti-mouse CD4, PE-conjugated anti-mouse CD8, or APC-conjugated anti-mouse B220 monoclonal antibodies (eBioscience). For CD4^+^ cell activation studies, splenocytes (5E^5^ per well) were stimulated in RPMI 1640+ 10% FBS with 5 µg/ml concanavalin A (Sigma–Aldrich) in 48-well plates for 72 h at 37°C. Following stimulation, cells were stained with FITC-conjugated anti-mouse CD4 and APC-conjugated anti-mouse CD25 monoclonal antibodies (eBioscience). All FACS analyses were conducted using a FACSCalibur (BD Biosciences).

### Histology

Paraffin cross-sections were made of gastrocnemius, heart and diaphragm muscles and stained with H&E. For gastrocnemius, images were analysed in Image J software (NIH) according to previously established methods (Spurney et al, 2009). For diaphragm, full tissue sections were scored for inflammation by a trained veterinary immunologist blinded to drug and genotype.

To assay fibrosis, paraffin embedded muscles were cross-sectioned and stained with Sirius Red. Tissue was imaged with a 4× objective, digital captures were made with Olympus software, and fibrotic signal quantified using Image J (NIH). Blood and background were removed from blinded images to prevent false detection of tissue and percent signals when threshold measurements were made during ImageJ quantitative analysis. The percentage fibrotic tissue was calculated as area reaching Sirius Red positive thresholds divided by total tissue area of the section.

### X-ray and microCT analysis of bone

Skeletons were harvested at trial endpoint and stored in 10% formalin. X-rays of tibias were obtained using a Cabinet X-Ray System (Faxitron Model 43855) with exposure at 50 kVp for 1.5 min. Magnification error was calculated to be ±0.02 mm. Images were scanned and tibia lengths measured in Adobe Illustrator (v6.0) at 2400% zoom. Measurements of the opposite tibia were also obtained physically with digital calipers during dissection, with results in agreement between methods. MicroCT analysis was performed on harvested femurs using a SkyScan 1172 MicroCT (Bruker, Belgium). Imaging was performed at 40 kV source voltage, 250 uA source current, 295 ms exposure time, and 0.4° rotation step, with a 0.5 mm aluminum filter. The imaging resolution size was 6.2 um. Three-dimensional reconstructions were performed with Skyscan NRecon and Dataviewer software. Trabecular bone was selected for analysis by a polygonal region of interest within the centre of femur, starting at 70 slices (0.43 mm) proximal from the growth plate and extending proximally 200 slices (1.23 mm) further. Trabecular measurements were obtained from 3D analysis of the selected bone using Skyscan CT-analyzer software.

**The paper explained**

PROBLEM:

Glucocorticoids have been a mainstay in medicine since their discovery over 60 years ago. They are powerful anti-inflammatory drugs used to treat a variety of conditions. However, due to a complex mechanism profile, glucocorticoids also cause harsh side effects such as brittle bones, muscle wasting, stunted growth, adrenal suppression and weight gain. Patients and doctors must therefore manage their net positive and negative effects. This is of particular importance in some chronic or paediatric disorders, where lifelong treatment is required and patients must live with serious side effects. DMD is a lethal genetic muscle disease for which glucocorticoids are the current standard of care. Though glucocorticoids produce established improvements in DMD patient outcome measures, their harsh side effects dramatically affect patients' quality of life. As a result, physicians typically delay treatment in young children until well after disease onset, and many families choose to stop treatment even though there is no alternative currently available in the clinic.

RESULTS:

The discovery that glucocorticoids possess several distinct sub-activities provides an intriguing opportunity to produce drugs that stimulate some of these activities while avoiding others. We discover VBP15 as a novel, orally administered compound that shares specific anti-inflammatory effects with glucocorticoids and also acts to stabilize cell membranes. Importantly, we also find that VBP15 avoids specific activities established to cause glucocorticoid side effects. Translating these findings into mice with muscular dystrophy, we find that both preventive and therapeutic regimens improve muscle strength and disease pathology. Further, this efficacy is displayed in the absence of hormonal, immunological and growth side effects seen in glucocorticoid treated mice.

IMPACT:

There is a clear need for improved treatments in chronic inflammatory diseases such as DMD, where safer drugs would improve quality of life and provide justification for neonatal screening. Data here confirms that small molecules can be produced which separate the sub-activities of glucocorticoids towards fulfilling this need. VBP15 is identified as the lead compound, which is actively being developed towards the clinic. Excitingly, proof-of-principle data shows that this compound provides efficacy in mice with muscular dystrophy while successfully eliminating important side effects. This provides new insight into glucocorticoid sub-activities, and demonstrates the potential to replace glucocorticoids as the standard of care for DMD as well as other chronic inflammatory diseases.

### Statistical analyses for animal trials

Unless otherwise noted, normality of each measurement was tested via Shapiro–Wilk normality test and normally distributed measurements were compared between *mdx* treatment groups using one-way ANOVA. Measurements that were not normally distributed were compared with a non-parametric test. For efficacy studies where *mdx* treatment comparisons showed a significant overall *p*-value, *post hoc* linear tests between each VBP15 dosage group and vehicle only were performed and resulting *p*-value adjusted for multiple testing by Sidak method. In side effect assays where comparisons showed a significant overall *p*-value, a Student's *t*-test was included between prednisolone and vehicle groups for normally distributed measurements.

## For more information

Muscular Dystrophy Association: http://mda.org/

Foundation to Eradicate Duchenne: http://duchennemd.org/

Parent Project Muscular Dystrophy: http://www.parentprojectmd.org/

Cooperative International Neuromuscular Research Group: http://www.cinrgresearch.org/

On preclinical DMD models: http://www.treat-nmd.eu/research/preclinical/dmd-models/

On standardized protocols for DMD model research: http://www.treat-nmd.eu/research/preclinical/dmd-sops/

## Supplementary material

As a service to our authors and readers, this journal provides supporting information supplied by the authors. Such materials are peer reviewed and may be re-organized for online delivery, but are not copy-edited or typeset. Technical support issues arising from supporting information (other than missing files) should be addressed to the authors.

**Review Process File**

**Figure S1.** Further *in vivo* efficacy data from the pre-symptomatic *mdx* trial. (A) The drop in force for EDL muscle after 10 lengthening contractions *ex vivo* showed a significant reduction upon VBP15 treatment at 15 mg/kg. (B and C) Live-animal imaging of cathepsin protease activity via ProSense680 shows reduced inflammatory disease in the forelimbs of VBP15-treated *mdx* mice (B) images; C quantitation of fluorescence; *n*≥6 mice/group). (D) H&E stained histology of diaphragm muscle shows a decrease in calcified myofibres upon VBP15 treatment. In diaphragm sections, calcified fibres were present in a majority (5 of 6) of vehicle *mdx* mice. VBP15 treated mice showed reduced calcification upon quantitation, with only 1–2 mice per group exhibiting any calcified fibres (*n* = 6 mice/group). (**p* < 0.05, *n* = 10 mice/group).

**Figure S2.** Further *in vivo* immunology and side effect data from the pre-symptomatic *mdx* trial. (A) Spleen to body mass ratios were measured upon trial conclusion (8 weeks). Note, *mdx* spleens were enlarged in comparison to WT, while VBP15 normalized spleen size toward WT levels and prednisolone reduced spleen size such that it was lower than WT. (B) Peripheral blood leucocyte counts were assayed at trial conclusion. (C–E) Histological analysis of gastrocnemii muscle shows increased skeletal muscle degeneration and fibrosis in prednisolone-treated mice, but not VBP15 mice. A significant increase in degenerating fibres (C) was present in prednisolone treated gastrocnemii, quantified in H&E stained sections. Digital quantitation (D) of sirius red staining shows a significant increase in skeletal muscle fibrosis in prednisolone treated mice. Representative images of sirius red staining are provided (E), to the right of the panel is a higher magnification image from the area outlined in box. (*n*≥12 mice/group for (A and B); *n* = 6 mice/group for (C and D); **p* < 0.05, ***p* < 0.05, ****p* < 0.05).
