# Supplementary material for: VBP15, a novel anti-inflammatory and membrane-stabilizer, improves muscular dystrophy without side effects
Source: EMBO Mol Med. 2013 Sep 9;5(10):1569–85. doi: 10.1002/emmm.201302621 (PMC3799580; doi:10.1002/emmm.201302621)
Supplement: Supplementary file 1 [file emmm0005-1569-SD1.pdf]

EMBO Molecular Medicine

VBP15, a novel anti-inflammatory and membrane-stabilizer, improves muscular dystrophy without hormonal side effects

Christopher R. Heier, Jesse M. Damsker, Qing Yu, Blythe C. Dillingham, Tony Huynh, Jack H. Van der Meulen, Arpana Sali, Brittany K. Miller, Aditi Phadke, Luana Scheffer, James Quinn, Kathleen Tatem, Sarah Jordan, Sherry Dadgar, Olga C. Rodriguez, Chris Albanese, Michael Calhoun, Heather Gordish, Jyoti K. Jaiswal, Edward M. Connor, John M. McCall, Eric P. Hoffman, Erica K. M. Reeves and Kanneboyina Nagaraju

Corresponding author: Kanneboyina Nagaraju, George Washington University

---

Review timeline:

Submission date:	08 February 2013
Accepted:	02 August 2013

Editor: Céline Carret

Transaction Report:

No Peer Review Process File is available with this article, as the authors have chosen not to make the review process public in this case.

© EMBO
