# Supplementary material for: VBP15, a novel anti-inflammatory and membrane-stabilizer, improves muscular dystrophy without side effects
Source: EMBO Mol Med. 2013 Sep 9;5(10):1569–85. doi: 10.1002/emmm.201302621 (PMC3799580; doi:10.1002/emmm.201302621)
Supplement: Supplementary file 2 [file emmm0005-1569-SD2.pdf]

Supporting Information:

VBP15, a novel anti-inflammatory and membrane-stabilizer, improves muscular dystrophy without hormonal side effects

Christopher R. Heier, Jesse M. Damsker, Qing Yu, Blythe C. Dillingham, Tony Huynh, Jack H. Van der Meulen, Arpana Sali, Brittany K. Miller, Aditi Phadke, Luana Scheffer, James Quinn, Kathleen Tatem, Sarah Jordan, Sherry Dadgar, Olga C. Rodriguez, Chris Albanese, Michael Calhoun, Heather Gordish, Jyoti K. Jaiswal, Edward M. Connor, John M. McCall, Eric P. Hoffman, Erica K. M. Reeves, and Kanneboyina Nagaraju

Table of Contents:

Supporting Information Figure 1: Further *in vivo* efficacy data from the pre-symptomatic *mdx* trial

Supporting Information Figure 2: Further *in vivo* immunology and side effect data from the pre-symptomatic *mdx* trial

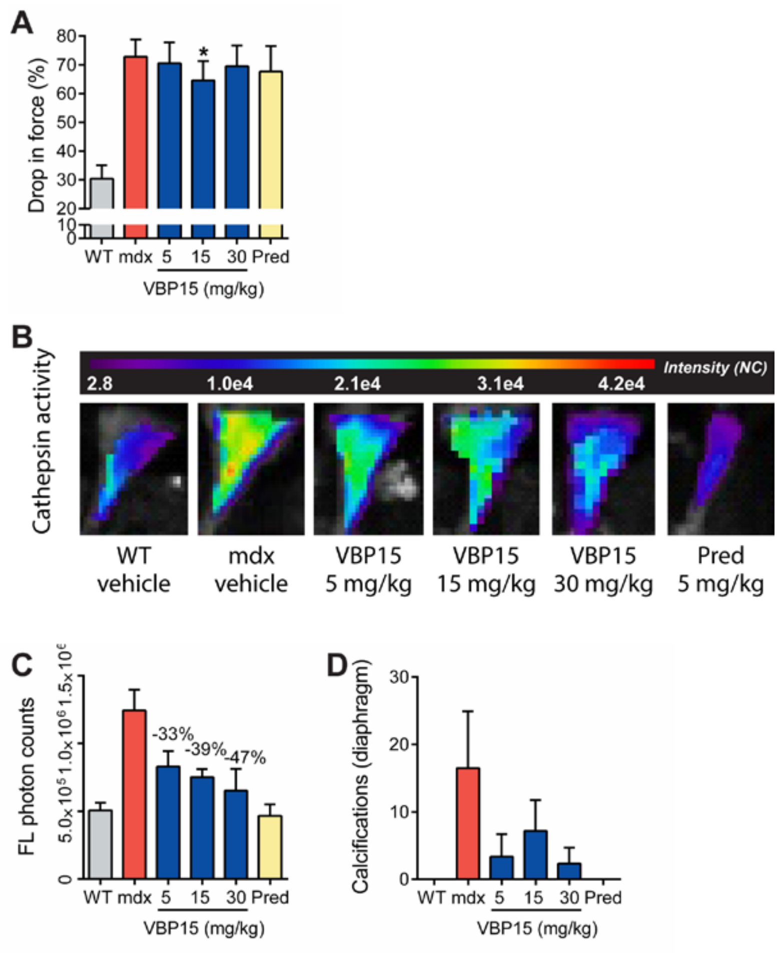

Supporting Information Figure 1. Further *in vivo* efficacy data from the pre-symptomatic *mdx* trial. A) The drop in force for EDL muscle after 10 lengthening contractions *ex vivo* showed a significant reduction upon VBP15 treatment at 15 mg/kg. **B-C**) Live-animal imaging of cathepsin protease activity via ProSense680 shows reduced inflammatory disease in the forelimbs of VBP15-treated *mdx* mice (**B** images; **C** quantitation of fluorescence; $n \geq 6$ mice/group). **D**) H&E stained histology of diaphragm muscle shows a decrease in calcified myofibers upon VBP15 treatment. In diaphragm sections, calcified fibers were present in a majority (5 of 6) of vehicle *mdx* mice. VBP15 treated mice showed reduced calcification upon quantitation, with only 1-2 mice per group exhibiting any calcified fibers (n = 6 mice/group). (*$p < 0.05$, n = 10 mice/group)

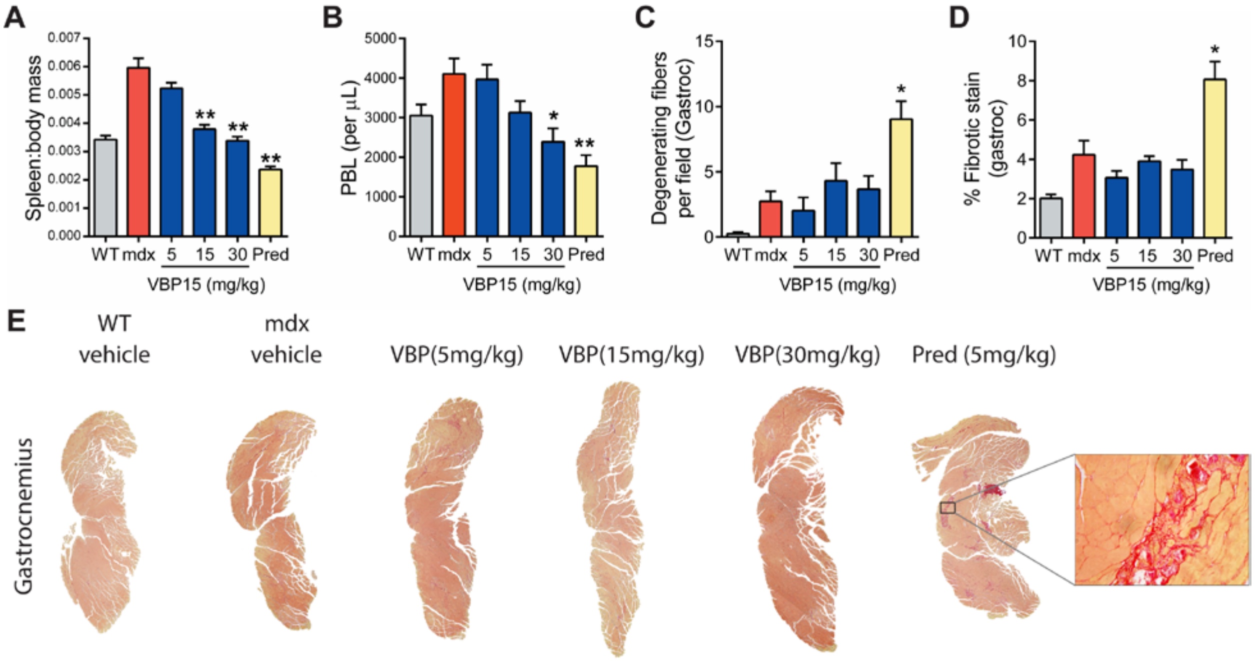

Supporting Information Figure 2. Further *in vivo* immunology and side effect data from the pre-symptomatic *mdx* trial. A) Spleen to body mass ratios were measured upon trial conclusion (8 wks). Note, *mdx* spleens were enlarged in comparison to WT, while VBP15 normalized spleen size toward WT levels and prednisolone reduced spleen size such that it was lower than WT. **B**) Peripheral blood leukocyte counts were assayed at trial conclusion. **C-E**) Histological analysis of gastrocnemii muscle shows increased skeletal muscle degeneration and fibrosis in prednisolone-treated mice, but not VBP15 mice. A significant increase in degenerating fibers (**C**) was present in prednisolone treated gastrocnemii, quantified in H&E stained sections. Digital quantitation (**D**) of sirius red staining shows a significant increase in skeletal muscle fibrosis in prednisolone treated mice. Representative images of sirius red staining are provided (**E**), to the right of the panel is a higher magnification image from the area outlined in box. (n ≥ 12 mice/group for **A,B**; n = 6 mice/group for **C,D**; *$p < 0.05$, **$p < 0.05$, ***$p < 0.05$)
